# Image smoothing method based on global gradient sparsity and local relative gradient constraint optimization

**DOI:** 10.1038/s41598-024-65886-5

**Published:** 2024-07-02

**Authors:** Siyuan Li, Yuan Liu, Jiafu Zeng, Yepeng Liu, Yue Li, Qingsong Xie

**Affiliations:** 1 School of Computer Science and Technology, Shandong Technology and Business University, Yantai, 264005 China; 2 Shandong Future Intelligent Financial Engineering Laboratory, Yantai, 264005 China; 3 Yantai Growth Drivers Conversion Research Institute and Yantai Science and Technology Achievement Transfer and Transformation Demonstration Base, Yantai, 264005 China; 4 Coal Economy Academy, Shandong Technology and Business University, Yantai, 264005 China

**Keywords:** Electrical and electronic engineering, Software

## Abstract

Removing texture while preserving the main structure of an image is a challenging task. To address this, this paper propose an image smoothing method based on global gradient sparsity and local relative gradient constraints optimization. To reduce the interference of complex texture details, adopting a multi-directional difference constrained global gradient sparsity decomposition method, which provides a guidance image with weaker texture detail gradients. Meanwhile, using the luminance channel as a reference, edge-aware operator is constructed based on local gradient constraints. This operator weakens the gradients of repetitive and similar texture details, enabling it to obtain more accurate structural information for guiding global optimization of the image. By projecting multi-directional differences onto the horizontal and vertical directions, a mapping from multi-directional differences to bi-directional gradients is achieved. Additionally, to ensure the consistency of measurement results, a multi-directional gradient normalization method is designed. Through experiments, we demonstrate that our method exhibits significant advantages in preserving image edges compared to current advanced smoothing methods.

## Introduction

Natural images typically encompass intricate texture details. The human visual system adeptly mitigates the influence of textures, prioritizing the extraction of crucial structural edge information from images for rapid comprehension. Similarly, the objective of image smoothing research is to attenuate texture details while retaining image structure edges to the greatest extent feasible. Smoothing constitutes a pivotal component in numerous image processing techniques, including image structure extraction, edge enhancement, and image recognition. Furthermore, the textures can be repurposed, for instance, in applications like image detail enhancement. Presently, diverse methods exist for achieving image smoothing. Based on distinct approaches to texture removal, image smoothing methods can be categorized into local filtering methods, global optimization methods, and deep learning methods.

Local filtering methods typically utilize sliding window techniques to achieve smoothing by recalculating the central pixel value of the window using the positional and value relationships among pixels within a local spatial neighborhood. Mean filtering accomplishes smoothing by substituting the central pixel with the average of the pixels in the local region. Although this method is straightforward, it often yields blurred edges in the smoothed image and escalates computational complexity with larger filter windows. Gaussian filtering enhances mean filtering by assigning varying weights to pixels in the filter window based on their distance from the central pixel and a Gaussian function equation. Pixels closer to the central pixel carry higher weights, while those farther away bear lower weights. Nonetheless, directly applying Gaussian filtering to images can also induce blurring of structural edges because Gaussian filtering treats all pixels in the image uniformly without discerning between structure and texture.

Bilateral filtering^1–4^ advances Gaussian filtering by incorporating both pixel value disparities and spatial offset distances between the central pixel and its neighboring pixels to determine filter weights. This methodology enables the preservation of edges while smoothing the image. However, images frequently contain pronounced gradient texture information, and solely incorporating pixel value domain variation may struggle to effectively address such images. The selection of parameters for bilateral filtering poses a challenge too. Augmenting the range weight might prove insufficient in mitigating strong gradient textures, while reducing it could result in edge blurring, thereby complicating the attainment of a harmonious balance between edge preservation and smoothing.

Guided filtering^5–8^ employs the fundamental concept of utilizing information from a guided image to adapt the filtering process and regulate the extent of image smoothing. The guided image can alternatively be the original input image itself. The relationship between the smoothed image and the guided image remains linear. This technique minimizes structural disparities between the local output image and the input image, thereby attaining optimal local results. Unlike the aforementioned local filtering methods, the size of the local window does not impact the algorithmic complexity of guided filtering, indicating substantial computational efficiency relative to other local filtering methods. While this filter excels at preserving edges during image smoothing, it may induce halos when confronted with intricate textures and details. Additionally, there exist methods^9–11^ that differentiate between the two by formulating metrics based on features observed in texture and edge regions, with the aim of achieving image smoothing objectives.

In comparison to local filtering methods, globally optimized methods founded on regularization constraints can simultaneously account for global variations in image gradients, thereby facilitating better preservation of overall consistency in smoothed images. Global optimization methods achieve smoothing by minimizing an energy equation, the equation typically comprising two components. One component ensures structural fidelity between the output and input images, quantified by the sum of pixel differences between corresponding pixels in the input and output images. The other component encompasses a regularization term, with various regularization terms employed by different methods. Nevertheless, the purpose of the regularization term is to categorize input pixels based on specified criteria and subsequently apply varying degrees of constraint to different pixels to achieve smoothing. Energy equations typically incorporate a smoothing parameter to modulate the influence of fidelity and regularization terms on the total energy of the equation.

Meyer et al.^12^ introduced a global optimization method based on total variation (*TV*) regularization. This approach solely relies on pixel gradients to differentiate textures from structural edges, often resulting in blurred edges or residual textures in the smoothed image. Furthermore, there exist methods that employ *L*-norm constraints to optimize images, such as $L_0$^13^, $L_1$^14^, and $L_2$^15^. Additionally, Du et al.^16^ proposed a texture-filtering method developed by integrating local Laplacian filters into joint filtering, thereby inheriting the advantages of joint bilateral filtering.

Zhou et al.^17^ introduced a filtering technique via iterative global optimization, demonstrating commendable scale-awareness and edge-preservation capabilities. Liu et al.^18^ proposed a novel global approach by embedding a bilateral filter into a least squares model, facilitating efficient edge-preserving smoothing. Golshan and Hasanzadeh^19^ devised a novel hysteresis smoothing method based on fuzzy norms. Wang et al.^20^ introduced a multi-matrix low-rank decomposition method for image denoising. Additionally, Liu et al.^21^ proposed a new iterative least squares method based on global optimization for efficient image edge preservation. Moreover, Liu et al.^22^ proposed image magnification techniques based on patch fitting. Building upon these advancements, Liu et al.^23^ presented an image smoothing method termed Histogram Equalization Content-Aware Patching and Direction-Constrained Sparse Gradient (HCDG). However, this method may produce seams in smoothed images with complex textures. Furthermore, Liu et al.^24^ introduced an algorithm that obtains texture information through user-clicked regions. Subsequently, Liu et al.^25^ proposed an image amplification method grounded on low-rank sparsity. Additionally, Liu et al.^26^ devised a non-convex and non-smooth optimization framework capable of achieving diverse and even contradictory smoothing behaviors via different parameter settings. Despite these advancements, the fixed pattern employed for texture removal and the absence of local region features may lead to artifacts or aliasing in the smoothed image. In addressing this, Sun et al.^27^ not only proposed an edge-guided filtering method but also designed post-processing techniques to optimize the filtered images.

With the advancement of neural network technology, numerous applications^28–30^ have undergone rapid development. However, the absence of a strict definition of smooth images has hindered the progress of image smoothing techniques using neural networks. It wasn’t until Convolutional Neural Networks (CNNs)^31^ became widely adopted that researchers began leveraging CNNs to learn filter kernels for enhancing the quality of image smoothing. Xu et al.^32^ introduced a deep edge-aware filter that trained the gradient domain of images within a deep CNN framework, learning a plethora of crucial edge-aware operators from data. This method provided a potent tool for approximating various filters without necessitating knowledge of the original model and details, achieving nearly a 200-fold acceleration compared to traditional filters. Lu et al.^33^ curated a large dataset by blending natural textures with pure structural images, utilized to train a Texture Prediction Network (TPN) combined with a Structure Prediction Network (SPN) to identify both components effectively. Zhu et al.^34^ constructed a dataset comprising 500 representative visual object categories and devised new loss functions on deep convolutional network architectures, yielding leading smoothing effects. Zhu et al.^35^ captured a multitude of image patches and extracted multimodal visual features to describe each patch. By training to capture edge block features and subsequently constructing a Gaussian mixture model for image smoothing, they achieved notable results. Xu et al.^36^ curated the Nankai Smoothing dataset (NKS), comprising 200 mixed structural-texture images generated by blending highly realistic vector images and images with natural textures. This process resembled the generation of noisy images in the field of image denoising. Liu et al.^26^ proposed a generalized framework for structure-preserving image smoothing. This framework utilized a truncated Huber penalty function, demonstrating strong flexibility under various parameter settings and achieving diverse smoothing properties.

**Figure 1:**
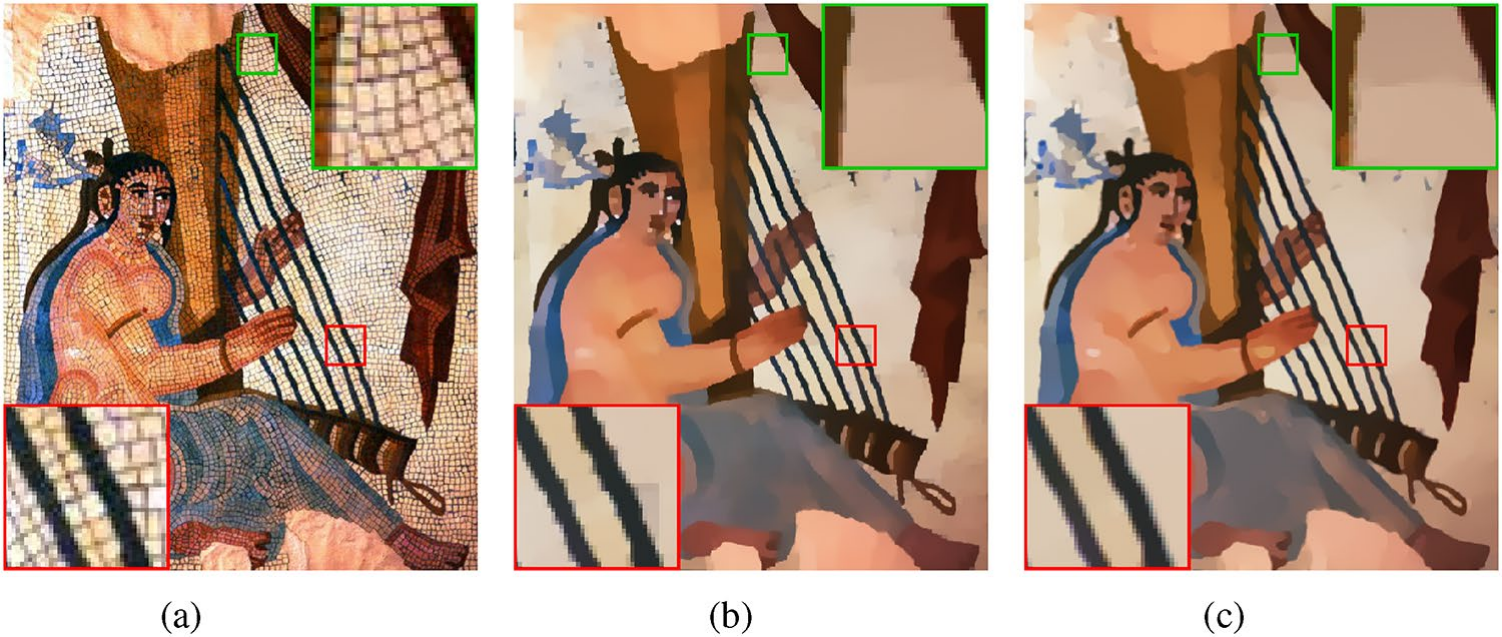
Comparison of advantages and disadvantages of different methods.

As shown in the Fig. 1, Fig. 1a is the input image, Fig. 1b is the image smoothed by relative total variation (RTV) method^15^, RTV method needs calculate the relative total variation to obtain the structural weight of the image, but the calculation of the relative total variation needs to use a large Gaussian filter, which will smooth out the edges with smaller gradients of the image, resulting in inaccurate structural weight. This eventually leads to the texture in region where strong gradient textures and edges cross is preserved as a structural edge, and jagged edges of the generated image, the red square in Fig﻿. 1b. Figure 1c shows the result after smoothing using the HCDG method^23^. In this method, the image is divided into different regions according to the density of structural edge and texture detail in the image, different regions are processed separately, and then, smooth the overall image. As shown in Fig﻿. 1c, the advantage of this is that the interference of the strong gradient texture on the whole image can be avoided, so that more structural edges with smaller gradients can be retained. The disadvantage is that when the texture of different regions in the image is quite different, there are some seams in the smoothed image due to the inconsistent smoothness of the method for different regions, shown in green square in Fig﻿. 1c.

To address these issues, this paper proposes a smoothing method based on global gradient sparsity and relative gradient constraint optimization. The method is built upon texture gradient suppression for global optimization smoothing, utilizing the $L_2$ norm as a regularization constraint. The $L_2$ norm of an image can be understood as the square root of the sum of squares of all pixels in the image. The $L_2$ norm possesses favorable mathematical properties such as differentiability, which facilitates solving optimization problems with the $L_2$ norm as a regularization term, often leading to global or near-global optimal solutions. Additionally, the $L_2$ norm preserves more image details and avoids producing step-like color transitions, as seen with the $L_0$ norm. This motivates the use of the $L_2$ norm as a regularization to constrain image optimization. This paper’s approach adopts a multi-directional difference constraint global gradient sparsity decomposition method, which obtains a smoothing component with weaker texture detail gradients through gradient sparsity. Meanwhile, it constructs an edge-aware operator using the gradient of the input image’s luminance channel as a reference. Compared to the aforementioned methods, this operator can more accurately capture image structural information, leading to excellent smoothing effects when employed as a constraint for guiding global image optimization. The main innovations are as follows:

1. By employing global image gradient sparse techniques, we diminish the high-frequency texture gradients in the image to obtain its smooth component.
2. Utilizing the log-luminance channel of the image combined with its smooth component to capture the structural weight of the image.
3. Designing a mapping relationship that incorporates multi-directional differences into two-directional gradient components.
4. Through the utilization of pixel structural weights to guide image optimization, suppressing pixel gradients in texture regions, thereby achieving the objective of smoothing the image.

## Methods

The method’s flowchart is illustrated in Fig﻿. 2. Firstly, this method specifies the input image to obtain its smoothing component, denoted as $I_s$. $I_n$ represents the texture gradient removed from the image through gradient sparsity, which can be obtained by pixel-wise subtraction of *I* and $I_s$. Then, it calculates the luminance channel of both the input image and its smoothing component. Based on these two luminance channels, the horizontal and vertical structural weights of the image can be computed. In regions of the input image characterized by edges, corresponding weights are smaller; conversely, in texture regions, weights are larger. Utilizing this information, we formulate a global optimization energy equation aimed at suppressing pixel gradients in texture regions, thereby achieving the objective of image smoothing. The subsequent sections of this chapter will systematically present our approach.

**Figure 2:**
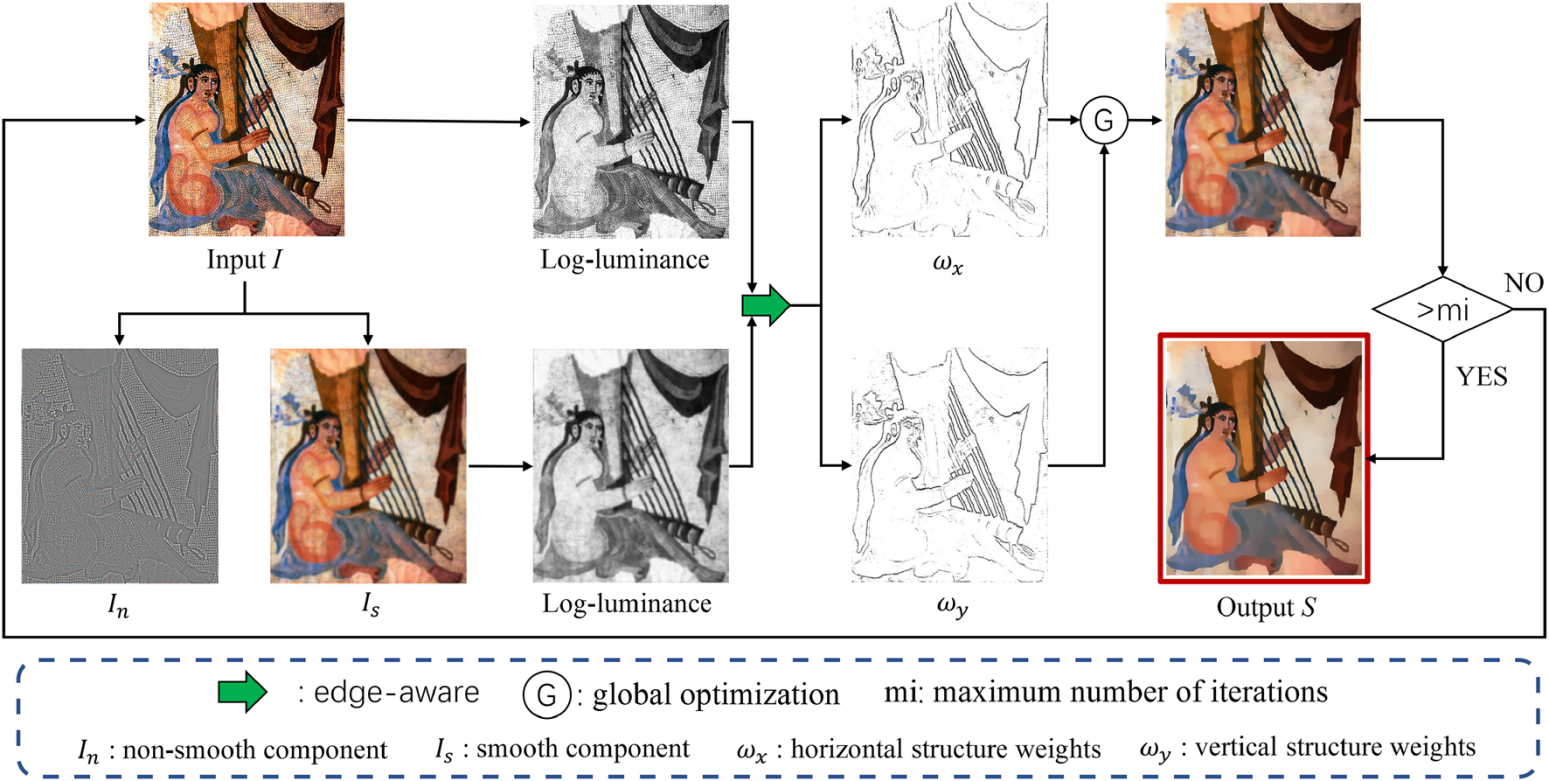
Flowchart of our method.

### Image gradient sparsity

The texture patches in the image exhibit similar and repetitive features. Utilizing gradient sparsity can remove the pixel gradient of the texture regions with high frequency while preserving the pixel gradient of the edge regions with low frequency as much as possible. The image after gradient sparsity is referred as the smooth component of the image. Therefore, it can be considered that the image *I* consists of two parts, can be expressed as:

1 $$\begin{aligned} I = I_s + I_n \end{aligned}$$

where $I_s$ represents the smooth component of the image, and $I_n$ corresponds to the texture gradients that has been removed. $I_s$ can be solved using multi-directional difference constraints, denoted as:

2 $$\begin{aligned} \begin{array}{c} \arg \min _{I_s}{\frac{1}{2}\Vert I-I_s \Vert _2^2}+\beta \cdot \sum _{d} \left\| \frac{\partial (I_s/g_\sigma )}{\partial _d} \right\| _2^2, \ d \in \{x,x+y,y,y-x \} \end{array} \end{aligned}$$

where $g_\sigma$ represents a mean filter of size $\sigma$ where all its values equal to 1. The smaller the value of $\sigma$, the sparser the resulting gradient, the symbol (/) represents the deconvolution operation, $\beta$ is the weight parameter used to control the smoothness of $I_s$, as the value of $\beta$ increases, the gradient matrix becomes sparser, leading to a smoother outcome. The regularization term $\frac{\partial (I_s/g_\sigma )}{\partial _d}$ consists of the first differences in $0^{\circ },45^{\circ },90^{\circ } \ \text {and} \ 135^{\circ }$ directions, which are $\{\partial _x, \partial _{x+y}, \partial _{y}, \partial _{y-x}\}$. Let’s set $I_s/g_\sigma = I_l$, then $I_s = g_\sigma \otimes I_l$, symbol $\otimes$ represents the convolution operation. Eq. (2)can be converted to the solution for $I_l$:

3 $$\begin{aligned} \begin{array}{c} arg\min _{I_l}{\Vert I-g_\sigma \otimes I_l \Vert _2^2}+\beta \cdot \sum _{d} \left\| \frac{\partial I_l}{\partial _d} \right\| _2^2, \ d \in \{x,x+y,y,y-x \} \end{array} \end{aligned}$$

Applying the matrix derivative formula to the above expression and setting it to zero yields:

4 $$\begin{aligned} \begin{array}{c} -2 * g_\sigma \otimes \left( I- g_\sigma \otimes I_l\right) +2 \beta \sum _{d}\partial _d^2I_l = 0, \ d \in \{x,x+y,y,y-x \} \end{array} \end{aligned}$$

by rearranging terms, $I_l$ represents as:

5 $$\begin{aligned} \begin{array}{c} I_l = \frac{g_\sigma \otimes I}{g_\sigma \otimes g_\sigma +\beta \sum _{d}\partial _d^2 }, \ d \in \{x,x+y,y,y-x \} \end{array} \end{aligned}$$

Given $I_s/g_\sigma = I_l$, we can solve for $I_s$. In order to speed up the operation, this paper use the Fast Fourier Transform (FFT) to accelerate it, which is expressed as:

6 $$\begin{aligned} I_s=g_\sigma \otimes {\mathscr {F}}^{-1}(\frac{{\mathscr {F}}(g_\sigma )*{\mathscr {F}}(I)}{{\mathscr {F}}(g_\sigma )*{\mathscr {F}}(g_\sigma )+\beta \sum _{d}^{} {\mathscr {F}}(\partial _d )*{\mathscr {F}}(\partial _d ) }) \end{aligned}$$

where ${\mathscr {F}}(\cdot )$ represents the FFT, ${\mathscr {F}}^{-1}(\cdot )$ represents the inverse FFT, $*$ denotes a complex conjugate operation, division $\frac{\cdot }{\cdot }$ is element-wise, $\partial _d$ represents the difference operator in the *d* direction, the difference operators corresponding to $\{x,x+y,y,y-x\}$ are $\{\{1, -1\}, \{0,1; -1,0\}, \{1; -1\}, \{1,0; 0,-1\}\}$.

### Log-luminance channel of the image

Many smoothing methods utilize the pixel means of the RGB channels as the image luminance. This paper opts for the luminance channel (*V*) in the HSV color space for the following reasons: Firstly, the *V* channel directly reflects the brightness level of the image, providing a more intuitive representation of the luminance information. Secondly, the *V* channel is color-independent, thus exhibiting higher stability and immunity to variations across different color channels. Moreover, the *V* channel facilitates a clearer distinction between luminance and color, enhancing effectiveness in image processing tasks. Lastly, the *V* channel demonstrates greater adaptability, effectively handling variations in illumination across different scenes. Therefore, employing the *V* channel as luminance contributes to improving the effectiveness and stability of image processing.

In the HSV color space, the *V* channel is used to represent the brightness information of the image. If we denote the pixel values of the three channels in the RGB space as R, G, and B, then the pixel values in the *V* channel can be calculated as follows:

7 $$\begin{aligned} V(I)_p = \max _{\phantom{a}}\{R_p,G_p,B_p\} \end{aligned}$$

where *I* is the input image, p denote each pixel.

Human perception of brightness is nonlinear, meaning that adding the same amount of light does not result in the same perceived increase in brightness. Therefore, to better represent changes in brightness, a logarithmic transformation can be used to map linear brightness changes into a nonlinear space. Logarithmic transformation ensures a more uniform distribution of brightness changes across the entire brightness range, resulting in more visually consistent brightness variations. Additionally, logarithmic transformation amplifies smaller brightness changes, thereby enhancing details in low-brightness areas of the image and reducing discontinuities in brightness changes caused by noise. This approach improves image contrast and visual quality. performing a logarithmic transformation on the *V* channel, the log-luminance channel of *I* can be represented as:

8 $$\begin{aligned} L(I)=\ln _{}{(1+k*V(I))} \end{aligned}$$

Where the constant 1 is introduced to prevent the presence of zero values in the input data, ensuring the transform function is defined across its entire domain. The parameter *k* is the contrast adjustment parameter, controlling the slope of the logarithmic transformation. A larger value of *k* results in a steeper slope of the logarithmic transformation, leading to more pronounced brightness changes and increased image contrast. Conversely, a smaller value of *k* yields a gentler slope of the logarithmic transformation, resulting in smoother brightness changes and reduced image contrast.

### Edge-aware operator

**Figure 3:**
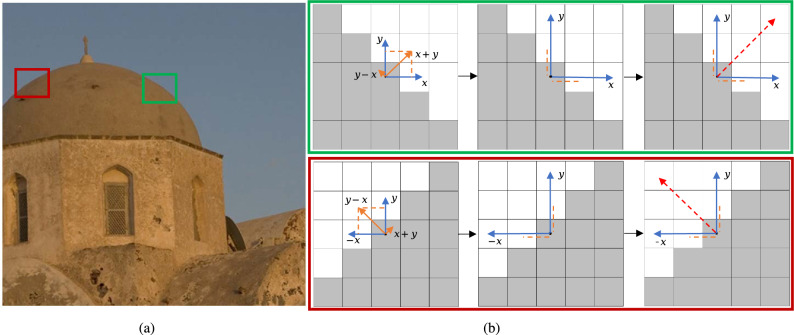
Multi-directional differential simulation of gradients. (**a**) Input. (**b**) Pixel gradient calculation.

First, we simulate pixel gradients using multi-directional differences. As shown in Fig. 3, we abstract the pixels in the green and red regions of the image Fig﻿. 3a into matrix representation, Fig﻿. 3b. The gray and white colors in the matrix represent two different pixel values. We first calculate the differences in four directions: $x,x+y,y$ and $y-x$. In the figure, blue and orange colors represent these differences. Since the $y-x$ difference is 0 in green box, it is not shown in the figure, but its principle is the same as the $x+y$ difference, shown in the red box. Then, projecting the $x+y$ difference (orange line) onto the *x* and *y* directions (blue lines), respectively. Finally, using the sum of the *x* and *y* direction vectors to simulate the gradient of that pixel.

Taking the horizontal direction as an example, a similar approach can be applied to the vertical direction as well. The horizontal gradient component of image *I* can be represented as:

9 $$\begin{aligned} \nabla _{x} I = \frac{ \vert \frac{\partial I}{\partial x} \vert +\cos 45^{\circ }\cdot \left( \vert \frac{\partial I}{\partial (x+y) } \vert +\vert \frac{\partial I}{\partial (y-x) } \vert \right) }{1+\cos 45^{\circ }+\cos 45^{\circ }} \end{aligned}$$

where *x* represents the horizontal direction, *y* represents the vertical direction, and the division is performed to ensure that the result is in the same range as the result obtained by directly using the difference in the *x* direction. Similarly, the vertical gradient component can be expressed as:

10 $$\begin{aligned} \nabla _{y} I = \frac{ \vert \frac{\partial I}{\partial y} \vert +\cos 45^{\circ }\cdot \left( \vert \frac{\partial I}{\partial (x+y) } \vert +\vert \frac{\partial I}{\partial (y-x) } \vert \right) }{1+\cos 45^{\circ }+\cos 45^{\circ }} \end{aligned}$$

The edge-aware operator can be expressed as:

11 $$\begin{aligned} \omega _d (I)=\left( \nabla _d L(I_s) \cdot \nabla _d L(I) +\varepsilon \right) ^{-1} \end{aligned}$$

where $I_s$ represents the smoothed component of *I* after gradient sparsity, $L(I_s)$ is its log-luminance channel. *L*(*I*) represents the log-luminance channel of *I*, $\nabla _d$ represents the gradient component of the image in the *d* direction, $\varepsilon$ is a very small value introduced to prevent division by zero, the division and multiplication operation is performed element-wise.

**Figure 4:**
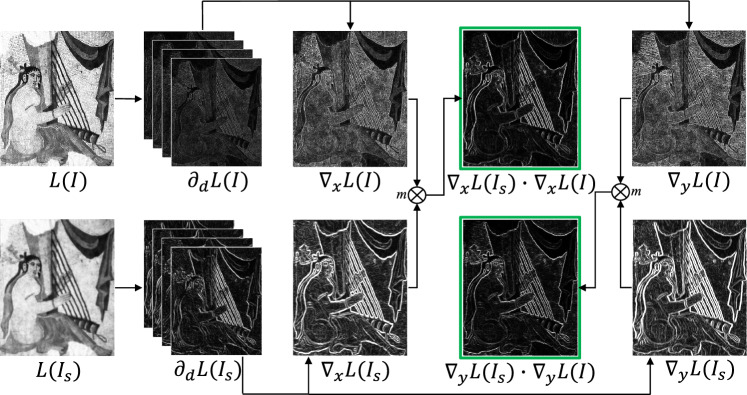
Flowchart of edge-aware, *L*(*I*) and $L(I_s)$ are the log-luminance channels of the input image and its smooth component, respectively.

This operator is capable of accurately recognizing the edge structures in the input image and serves as weight parameters to guide optimization. The flowchart is shown in Fig. 4.

After obtaining $\nabla _d L(I_s) \cdot \nabla _d L(I)$, it needs to be inverted. To prevent division by zero, a small constant $\varepsilon$ is added to it. Besides its role in preventing division by zero, $\varepsilon$ also impacts the sharpness of the final smoothed image.

### The scheme of global optimization

Our method is based on the idea of gradient suppression, which can obtain the smooth effect by limiting the gradient of the pixels in the non-edge region of the image. This method achieve smoothing by minimizing our energy equation, which can be expressed as follows:

12 $$\begin{aligned} \arg \min _{S} \left\| S-I \right\| _2 ^{2}+\lambda \sum _{p}^{} \left( \omega _{x,p}(I) \left( \frac{\partial S }{\partial x } \right) _p^2 +\omega _{y,p}(I) \left( \frac{\partial S }{\partial y } \right) _p^2 \right) \end{aligned}$$

This equation is divided into two parts, fidelity term $\left\| S - I \right\| _2 ^2$ and constraint term $\omega _{x,p}(I) \left( \frac{\partial S }{\partial x } \right) _p^2 +\omega _{y,p}(I) \left( \frac{\partial S }{\partial y } \right) _p^2$. *p* represents each pixel. The fidelity term measures the similarity between an input image *I* and a smoothed output image *S* by computing the difference between the pixels in the two images. $\omega _{}(I)$ is the edge-aware operator we designed. The parameter $\lambda$ serves as the global smoothing parameter, controlling the balance between the two components of the equation. When the $\lambda$ is small, the fidelity term will get a large weight in minimizing this energy equation, which forces the output image *S* to be more similar to the input image *I*. Conversely, when the $\lambda$ is large, the constraint term gets more weight and the output image will be smoother. It is worth noting that we express Eq. (12) in pixels for the sake of understanding the principle of image smoothing. However, in practical calculations, we perform computations on a image basis for ease of operation and to improve computational speed.

**Algorithm 1:**
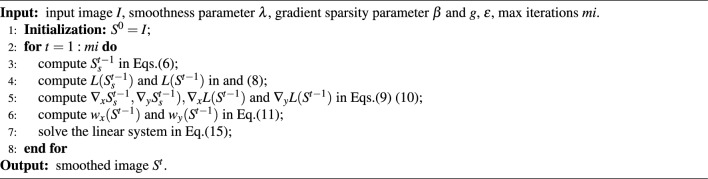
Smooth method

Based on the previous description, $I_s(I)$ and *L*(*I*) can be solved using Eqs. (6) and (8). Then, $w_x$ and $w_y$ can be solved using Eq. (11). Therefore, Eq. (12) can be represented as follows:

13 $$\begin{aligned} arg\min _{S} \left\| S-I \right\| _2^2+\lambda \sum _{d\in \{x,y\}}^{} \left\| \omega _d(I)\cdot \frac{\partial S }{\partial d }\cdot \frac{\partial S }{\partial d } \right\| _1 \end{aligned}$$

where the operation $(\cdot )$ denotes matrix element-wise multiplication.

Like many other methods, our method also requires multiple iterations to obtain the satisfactory result. Here is our iterative scheme, rewritten in matrix form:

14 $$\begin{aligned} \left( v_{S^t}-v_{S^{t-1}} \right) ^{T} \left( v_{S^t}-v_{S^{t-1}} \right) +\lambda \left( \sum _{d\in \{x,y\}}^{} v_{S^t} ^TC_d ^TW_dC_d ^Tv_{S^t} ^T\right) \end{aligned}$$

where $S^{t-1}$ is input image, $S^{t}$ is output image, $v_{S^{t-1}}$ and $v_{S^t}$ denote their respective vector representations. *t* is an integer greater than or equal to 1, $S^0 = I$, *T* represents the matrix transpose operation, $W_x$ and $W_y$ are diagonal matrices that incorporate the edge-aware operators $\omega _x$ and $\omega _y$, $C_x$ and $C_y$ are the Toeplitz matrices from the discrete gradient operators with forward difference. The vector $v_{S^t}$ that minimizes Eq. (14) is uniquely defined as the solution of the linear system, Taking the derivative of Eq. (14) with respect to $v_{S^t}$ and setting the result to zero, represent as follows:

15 $$\begin{aligned} v_{S^{t}}=\left( 1+\lambda \sum _{d\in \{x,y\}} C_d ^TW_dC_d\right) ^{-1}v_{S^{t-1}} \end{aligned}$$

where 1 is an identity matrix and $C_x ^TW_xC_x+C_y ^TW_yC_y$ is the weight matrix computed based on the input image $v_S^{t-1}$, $(1+\lambda \sum _{d\in \{x,y\}} C_d ^TW_dC_d)$ is the symmetric positive definite Laplacian matrix. The optimized result is used as the input for subsequent calculations, leading to satisfactory smoothing effects after multiple iterations. The complete algorithm flow is summarized in Algorithm 1.

## Result and discussion

In order to prove the effectiveness of our method, we conducted comparative experiments with the following methods. Global optimization methods are TV^37^, RTV^15^, DSG^38^, ILS^21^, muGIF^8^, and HCDG^23^, deep learning method are VDCNN^34^ and ResNet^34^. These methods have gained widespread recognition in the field of image smoothing, representing the direction of development in image smooth area.

### Ablation experiment

To demonstrate the superiority of our method in edge detection, we compared the gradients of the smoothed component after sparsifying image gradients with those of the original image. Compared to previous methods such as TV method, our approach overcomes interference from strong gradient textures, thus emphasizing the prominent structural edges in the image.

**Figure 5:**
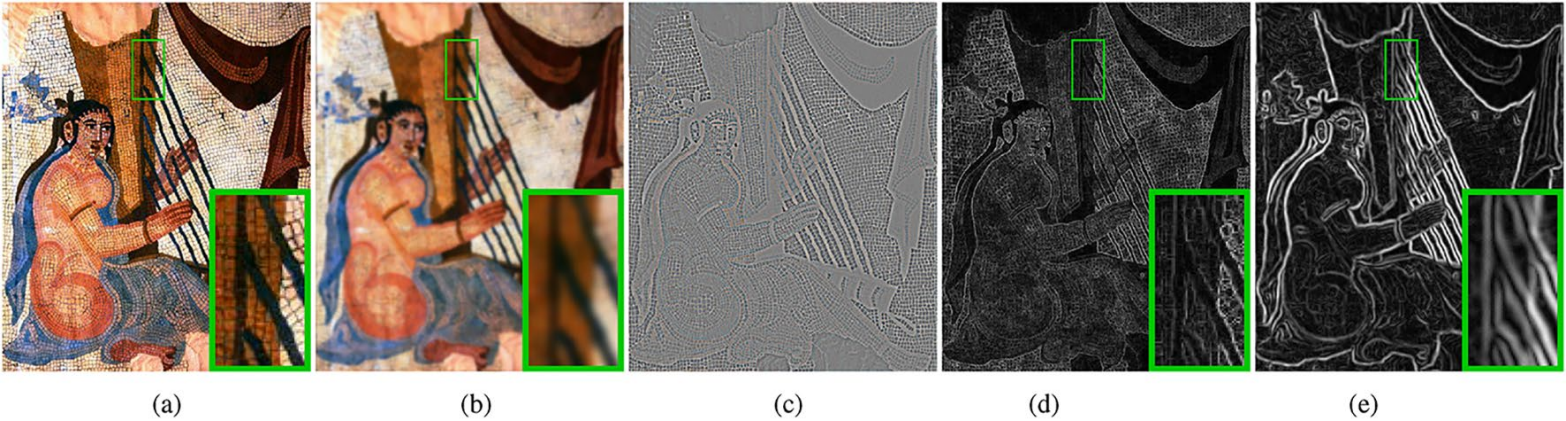
Gradient sparsity. (**a**) I. (**b**) Is. (**c**) In. (**d**) gradient of I. (**e**) gradient of Is.

We perform gradient sparsity on image *I* with parameter $\beta =150,\sigma = 4$, as shown in Fig﻿. 5. Where, Fig﻿. 5a represents the input image *I*, while Fig﻿. 5b represents the smooth component of *I*. Figure 5c represents the high-frequency texture gradient information that has been removed. By comparing the gradient images of *I* and $I_s$, i.e. Fig﻿. 5d and e, it is evident that $I_s$ can better highlight the structural information in the image. For example, in the green box region, it is difficult to discern the boundary area of the harp strings in Fig﻿. 5d, but it is clearly visible in Fig﻿. 5e.

### Texture Images with artificial blending

In order to verify that our method can preserve the edges of the image as much as possible while removing the textures. We conduct experiments using the Nankai Smoothing (NKS) dataset proposed by Xu et al.^36^. The author selected 20 highly realistic vector images from thousands of vector images containing free structures. For example, the images depicted in Fig﻿. 6a. Additionally, 10 natural texture images were chosen such as Fig﻿. 6b. These two sets were blended according to a structural ratio ranging from 0.7 to 0.85, resulting in visually natural texture-structure composite images, Fig﻿. 6c. Compared to other image smoothing datasets, NKS comes with a ground truth. As the ground truth are smooth images, it becomes easier to identify structural edges, making it possible for the smoothed images to closely match the ground truth without texture residue. Therefore, the better the edge-preserving smoothing effect, the closer the structural edges of the smoothed images align with the ground truth.

**Figure 6:**
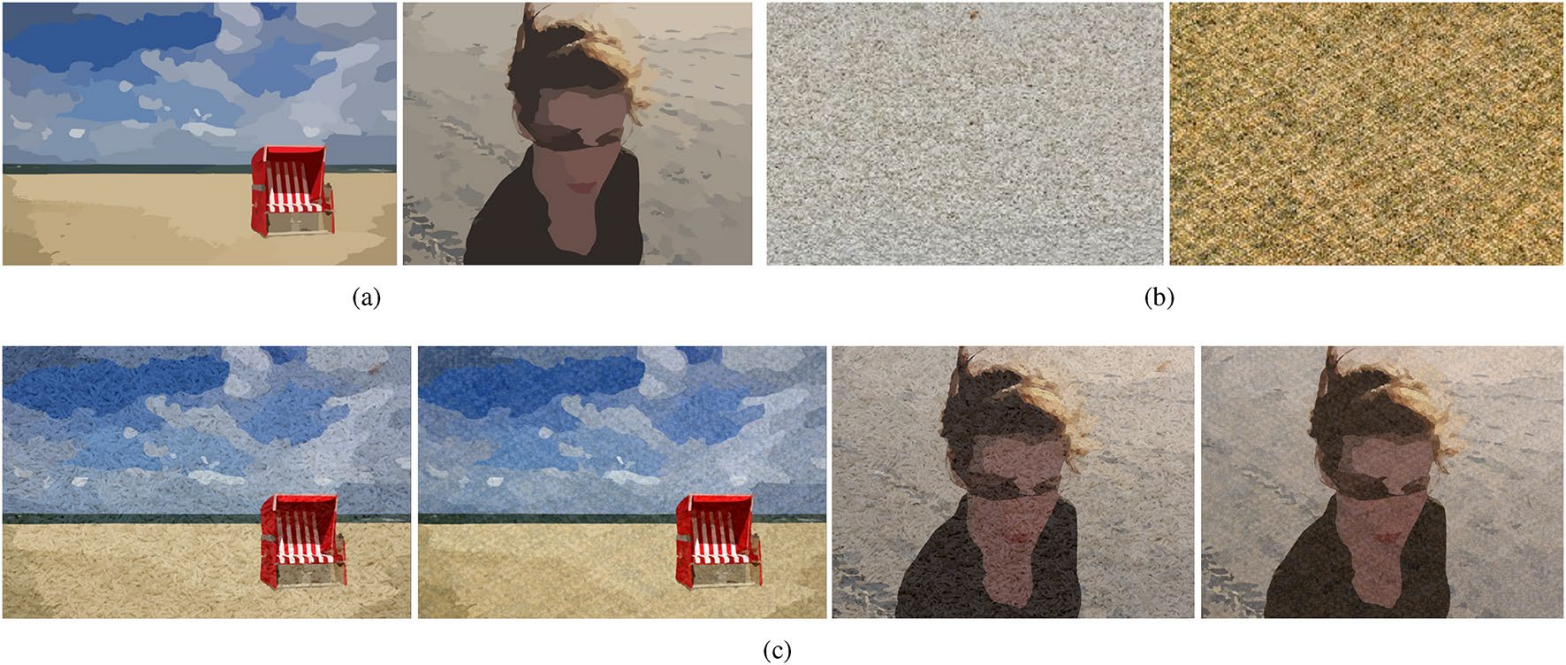
(**a**) Smooth vector images from NKS^36^. (**b**) Texture images from NKS^36^. (**c**) Artificially blended texture-structure images from NKS^36^.

**Figure 7:**
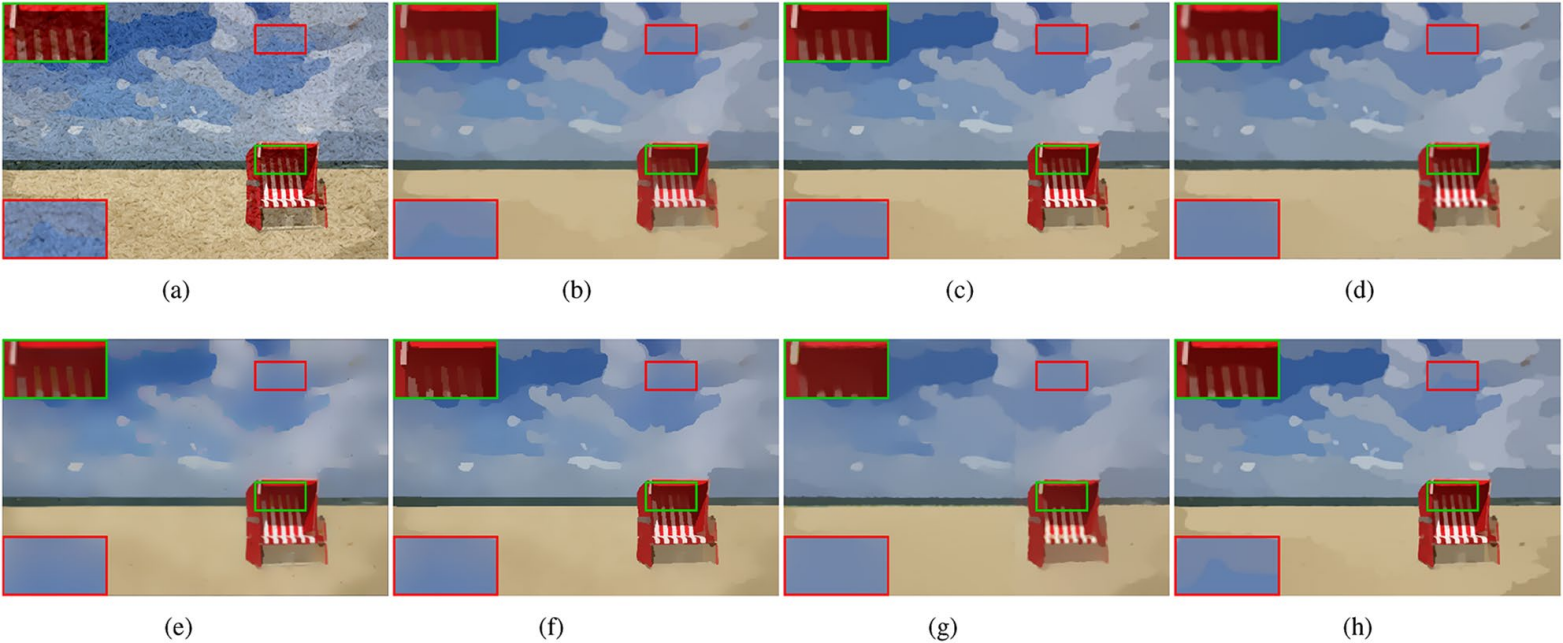
(**a**) Input image from NKS^36^; (**b**) TV ($\lambda =0.2$; (**c**) RTV ($\lambda =0.004, \; \sigma =3$); (**d**) DSG ($\lambda =0.008, \; k=50$); (**e**) ILS ($\lambda =8, \; \gamma =6/255$) (**f**) muGIF ($\lambda =0.015$); (**g**) HCDG($\lambda _d=38, \; \lambda =0.08$); (**h**) ours ($\beta =100, \; g=4, \; \lambda =0.005$).

**Figure 8:**
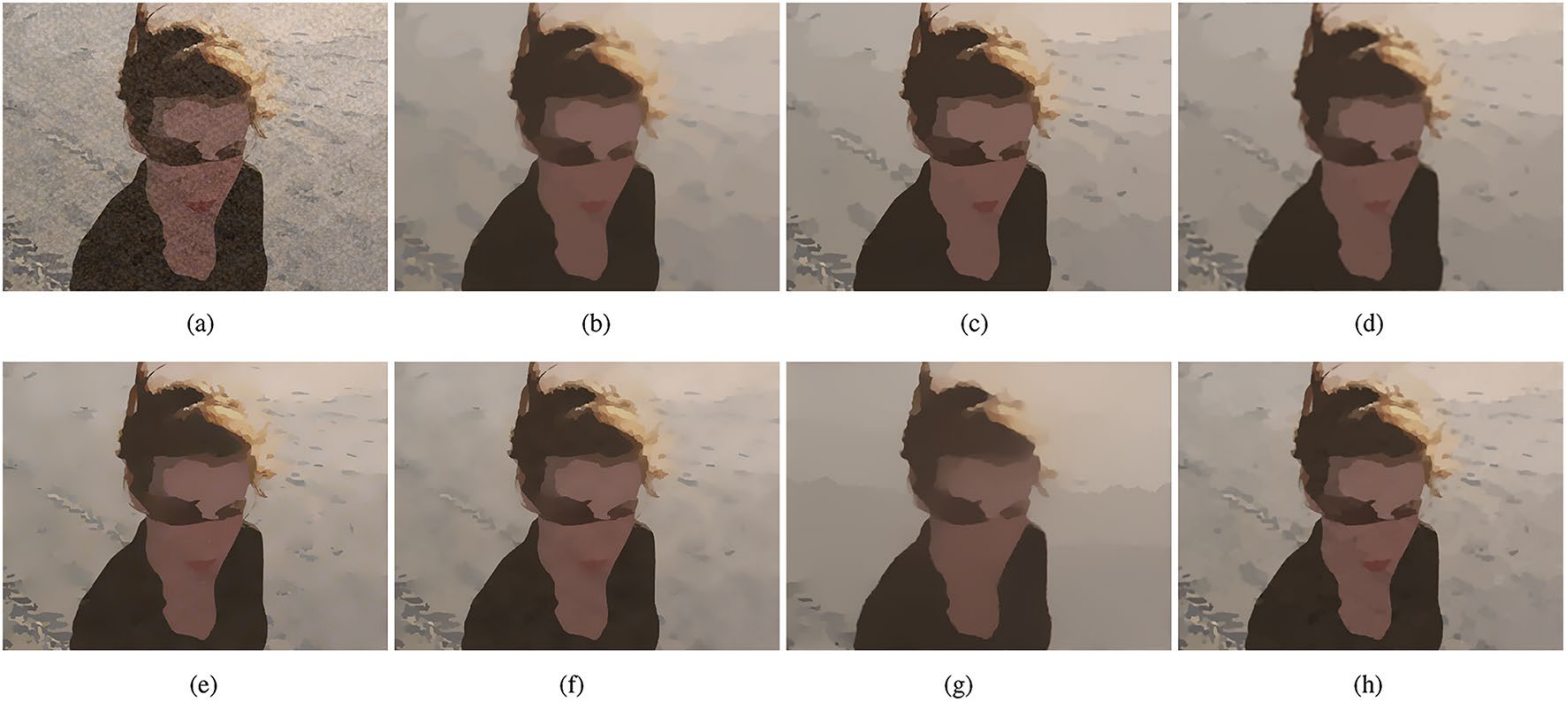
(**a**) Input image from NKS^36^; (**b**) TV ($\lambda =0.15$); (**c**) RTV ($\lambda =0.002, \; \sigma =3$); (**d**) DSG ($\lambda =0.005, \; k=50$); (**e**) ILS ($\lambda =10, \; \gamma =5/255$) (**f**) muGIF ($\lambda =0.005$); (**g**) HCDG ($\lambda _d=38, \; \lambda =0.05$); (**h**) ours ($\beta =100, \; g=4, \; \lambda =0.0015$).

As shown in Fig﻿s. 7, 8, we picked two images , Fig﻿s. 7a and 8a, with texture from the NKS. Through experiments we found that the comparison methods selected have excellent texture removal effects, but there are some problems with edge preservation. The TV method removes some fine structural edges, such as the beach chair in Fig﻿. 7b and the creases on the ground in Fig﻿. 8b. RTV method, Figs. 7c and 8c, removed part of the edges exist in ground truth, such as the edges of the cloud in Fig﻿. 7c and the shadow of the beach in Fig﻿. 8c. The images, Fig﻿s. 7d and 8d, smoothed by method DSG also has a loss of details compared with ground truth. The image smoothed by ILS method removed many structure edges, as shown in Fig﻿s. 7e and 8e. muGIF method , Fig﻿s. 7f and 8f, has the same problem, the image smoothed by muGIF removed many edges of the cloud and white stripes of the beach chair in in Fig﻿. 7f. HCDG method, Fig﻿s. 7g and 8g lose a lot of structural edge information. Our method can still preserve complete edge details after removing artificially added textures, as shown in Fig﻿s. 7h and 8h, edges of the clouds and white streaks of the beach chair in Fig﻿. 7h and shadow of the beach in Fig﻿. 8h have been preserved.

### Images with strong gradient textures

In this subsection, we will verify the texture removal ability of our method. The images we selected in this subsection contain a lot of strong gradient textures, as shown in Fig﻿s. 9, 10, 11 and 12, These strong gradient textures are very similar to the image structure edges, which tests the method’s ability to recognize the image structure edges.

**Figure 9:**
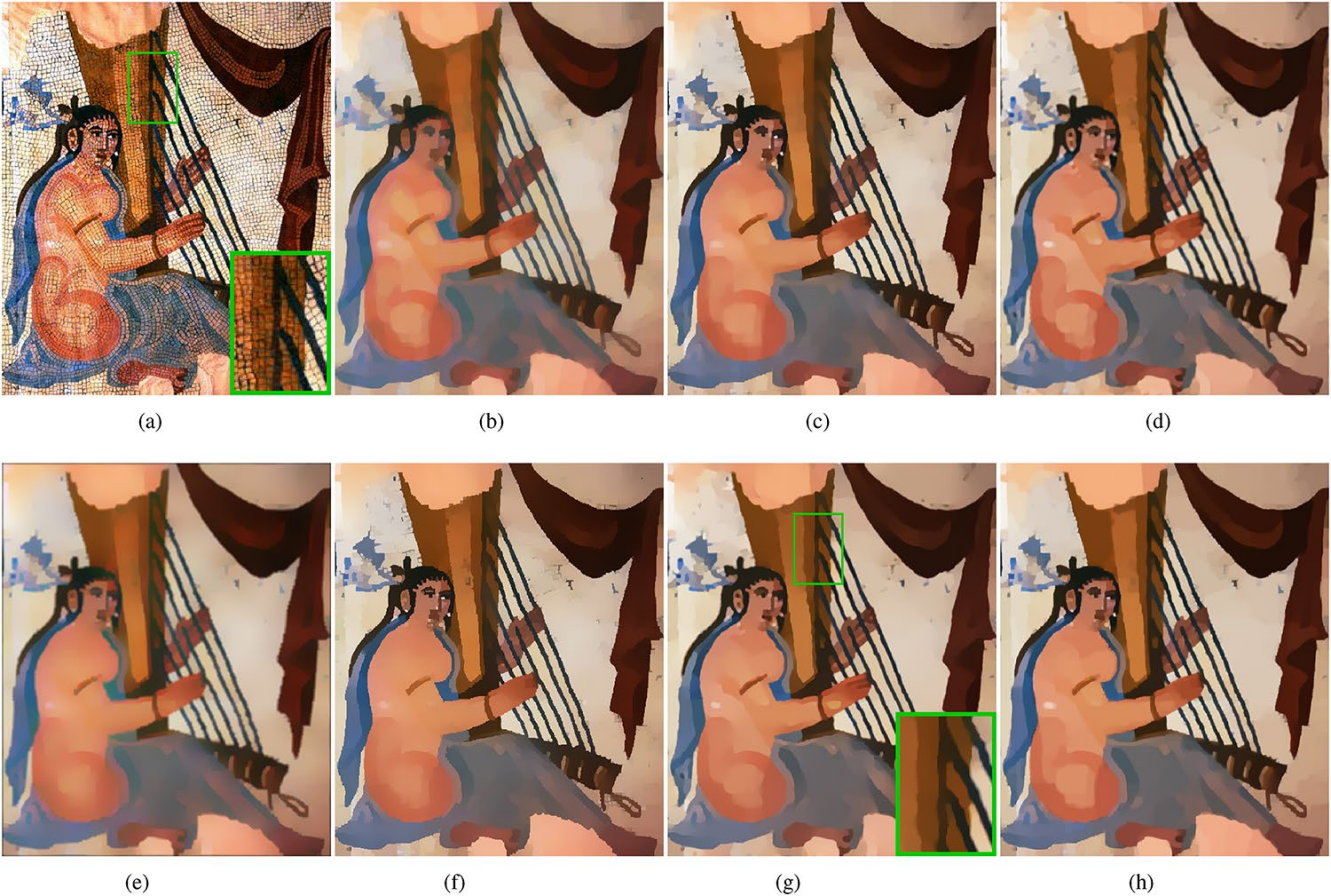
(**a**) Input; (**b**) TV ($\lambda =0.4$); (**c**) RTV ($\lambda =0.015, \sigma =3$); (**d**) DSG ($\lambda =0.015, \;k=50$); (**e**) ILS ($\lambda =10, \;\gamma =4/255$) (**f**) muGIF ($\lambda =0.04$); (**g**) HCDG ($\lambda _d=38 \;\lambda =0.04$); (**h**) ours ($\beta =200 \;g=4, \; \lambda =0.015$).

**Figure 10:**
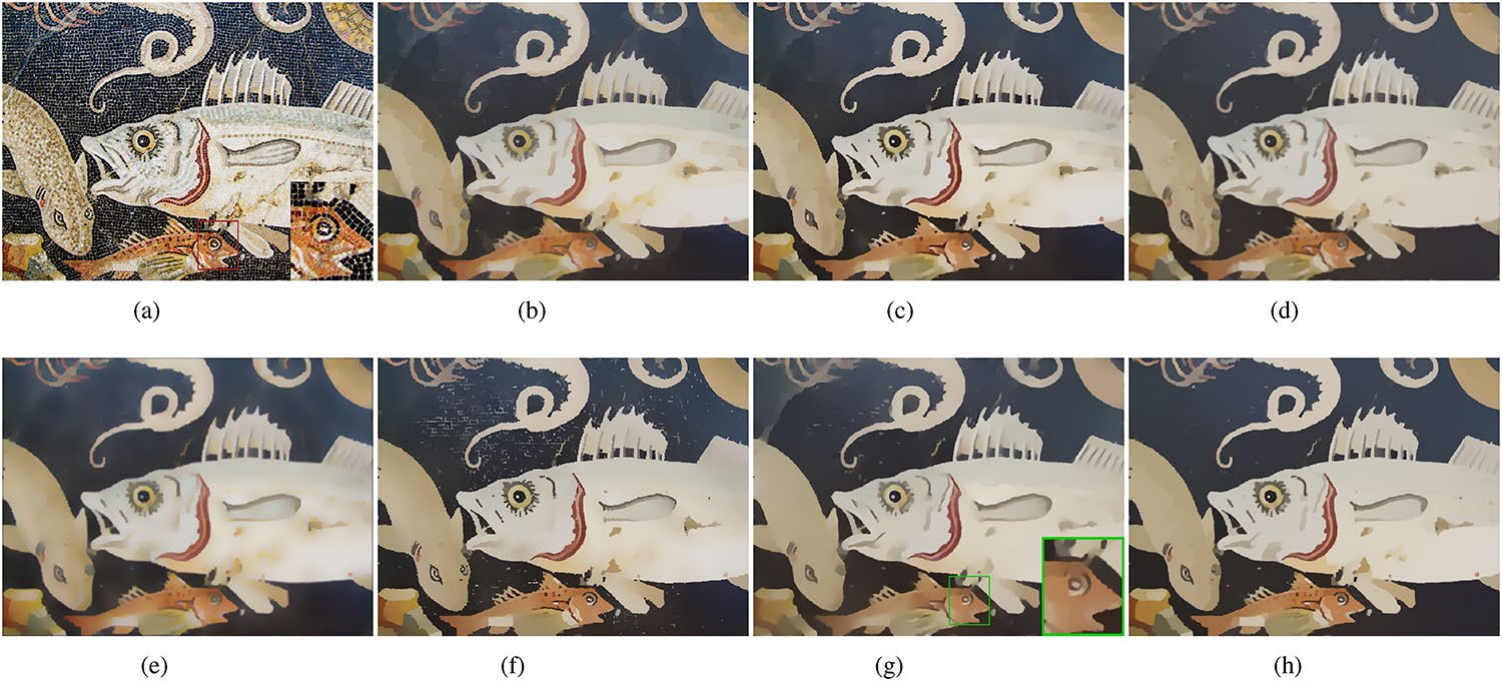
(**a**) Input; (**b**) TV ($\lambda =0.5$); (**c**) RTV ($\lambda =0.02, \;\sigma =5$); (**d**) DSG ($\lambda =0.03, \;k=200$); (**e**) ILS ($\lambda =10, \gamma =4/255$) (**f**) muGIF ($\lambda =0.08$); (**g**) HCDG ($\lambda _d=55, \; \lambda =0.2$); (**h**) ours($\beta =200, \; g=5, \; \lambda =0.03$).

**Figure 11:**
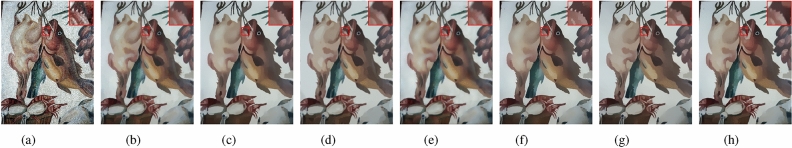
(**a**) Input; (**b**) TV ($\lambda =0.2$); (**c**) RTV($\lambda =0.015, \; \sigma =3$); (**d**) DSG ($\lambda =0.01, \; k=100$); (**e**) ILS ($\lambda =5, \; \gamma =4/255$) (**f**) muGIF ($\lambda =0.02$); (**g**) HCDG ($\lambda _d=38, \; \lambda =0.03$); (**h**) ours ($\beta =30, \; g=4, \; \lambda =0.015$).

**Figure 12:**
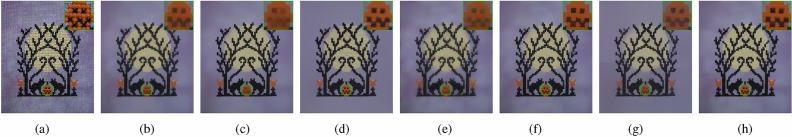
(**a**) Input; (**b**) TV ($\lambda =0.35$); (**c**) RTV ($\lambda =0.02, \; \sigma =3$); (**d**) DSG ($\lambda =0.015, \; k=200$); (**e**) ILS ($\lambda =10, \; \gamma =8/255$) (**f**) muGIF ($\lambda =0.02$); (**g**) HCDG ($\lambda _d=38, \; \lambda =0.1$); (**h**) ours ($\beta =160, \; g=4, \; \lambda =0.015$).

The smoothed images, Fig﻿s. 9b and 10b, by TV method removed most of the texture, but the smoothed image has color cast compared with the input image, such as the harp strings in Fig﻿. 9b. As shown in Fig﻿s. 9c and 10c, the smoothed image by RTV method not only removes the texture but also removes some weak gradient edges, such as the overlap region between the harp and the harp strings in Fig﻿. 9c and the eye of the little fish in Fig﻿. 10c. The image, Fig﻿. 9c, smoothed by DSG method preserved the weak gradient edge, but the structural edges are not sharp enough and some textures remains. In Fig﻿. 10d, the DSG method still fails to preserve the eye contour of the little fish. ILS method needs to use small scale Gaussian filter to pre-smooth the image with strong gradient texture, which will undoubtedly reduce the gradient of the structural edges. As shown in Fig﻿. 9e and 10e, the edges of the processed image will be blurred and have color difference. The problem with the muGIF method is that it can’t accurately distinguish between image structure edges and strong gradient textures, as shown in Fig﻿. 9f, some structural edges in the overlap region between the harp and harp strings are removed, while some texture remains on the walls, in Fig﻿. 10f, this method preserved the eye contour of the little fish, and there is still more textures in the blue water. The HCDG method, Fig﻿s. 9g and 10g, has a better performance, however, in the smoothed image, Figure 9(g), there is texture remaining and the eye contour of the fish is blurred. As shown in Fig﻿s. 9h and 10h, our method has ability to remove most texture, only a few texture remains, and on this basis, our method can preserve most of the structural edges. In Fig﻿s. 11 and 12, our method effectively removes the texture from the images while preserving weak gradient edges and small-scale structural edges in the smoothed images. For example, the teeth of the fish in Fig﻿. 11h and the facial features of the pumpkin in Fig﻿. 12h are retained. We have zoomed in on these areas for ease of observation.

### Comparison with deep learning methods

In this section, we compare our method with deep learning methods, specifically ResNet^34^ and VDCNN^34^. We retrain the models based on their papers and fine-tune the hyper parameters according to the actual performance of the models. The experimental results are presented in Fig﻿s. 13 and 14.

**Figure 13:**
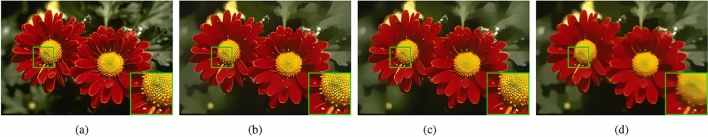
(**a**) Input; (**b**) ResNet; (**c**) VDCNN; (**d**) ours ($\beta =150, \; g=5, \; \lambda =0.005$).

**Figure 14:**
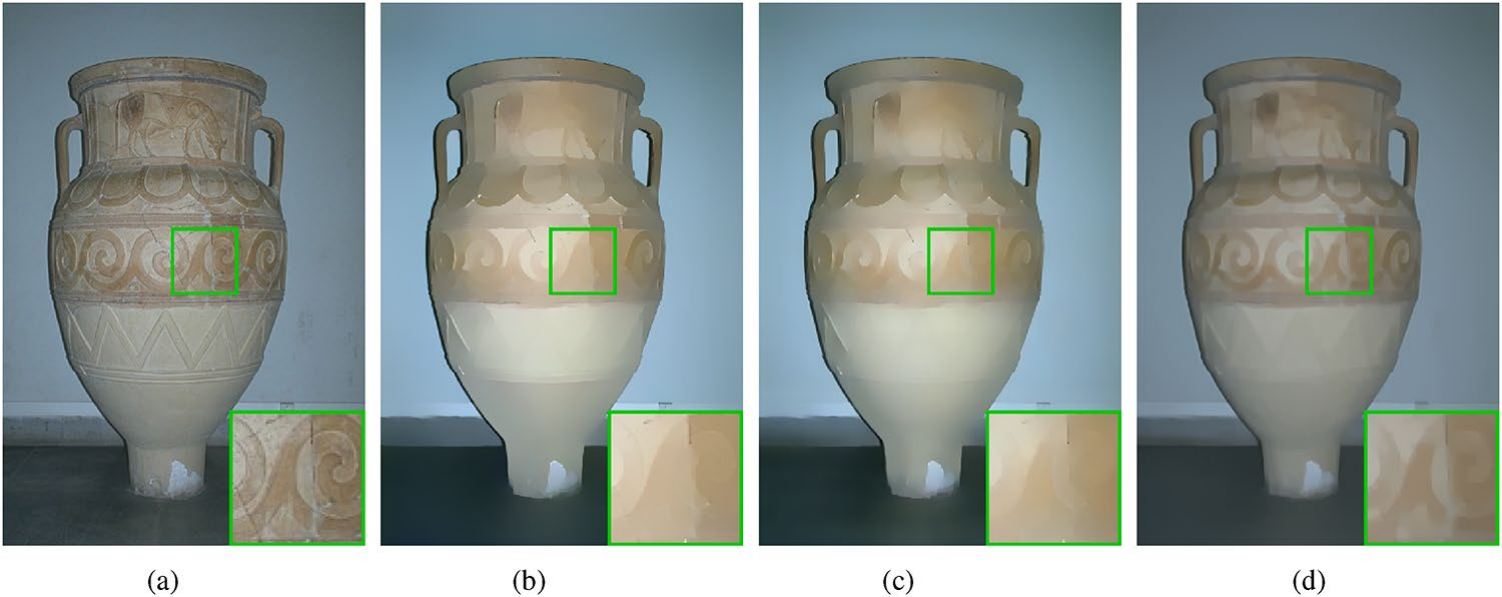
(**a**) Input; (**b**) ResNet; (**c**) VDCNN; (**d**) ours ($\beta =150, \; g=5, \; \lambda =0.001$).

From Fig﻿. 13, it is evident that both the ResNet model and the VDCNN model, Fig﻿s. 13b and c, fail to smooth out the yellow flower center. While our method effectively smooths the flower center, it also preserves weak edges in the image, such as the overlapping edges of the petals. In Fig﻿. 14, the results of the ResNet model and VDCNN model both exhibit color shifts, as shown in Fig﻿. 14b and c respectively. Compared to the input image, there is a noticeable increase in brightness in their outputs. The results produced by deep learning methods are uncontrollable, and thus, this issue cannot be addressed by adjusting hyper parameters. Our method generates more stable results, as shown in Fig﻿. 14d, where the smoothed result closely matches the input image in terms of color. Additionally, the texture pattern on the vase is preserved during smoothing, demonstrating excellent smoothing results.

### Numerical verification

To further validate the effectiveness of our method, we conducted an analysis of the experimental results using Structural Similarity (SSIM), Peak Signal-to-Noise Ratio (PSNR), and Information Entropy (IEP). PSNR and SSIM are commonly used in papers related to image enlargement and denoising, they can measure the similarity between two images from different perspectives. Unlike these image processing techniques, image smoothing lack a ground truth for comparison. Therefore, in most articles, people compare textured input images with smoothed images. However, the problem is that if some texture that should have been removed is still present, this can similarly lead to an increase in PSNR and SSIM, and therefore needs to be combined with a visual image as a reference. In the case where the visual texture is completely obliterated, a higher PSNR and SSIM means that the image retains a more complete edge structure. We calculated the PSNR and SSIM values between the input images and the smoothed images, and the results are summarized in Table 1. In the rows corresponding to SSIM and PSNR, the values in bold represent the maximum values in each row. From the Table 1, it can be seen that our method performs excellently in most cases.

**Table 1:** PSNR, SSIM and IEP compare.

		Input	TV	RTV	DSG	ILS	muGIF	HCDG	Ours
Figure 7	PSNR	–	31.412	31.6454	30.9223	30.8071	31.2419	30.3627	**31.6592**
	SSIM	–	0.8888	0.8951	0.8747	0.8709	0.8858	0.861	**0.8953**
	IEP	7.1441	6.2687	6.3032	6.3101	6.7182	6.6101	6.4965	*6.2389*
Figure 8	PSNR	–	32.7837	33.285	32.6398	33.5445	33.3602	31.2363	**34.0748**
	SSIM	–	0.8221	0.8371	0.8163	0.853	0.8388	0.7921	**0.8538**
	IEP	7.2339	6.471	*6.2478*	6.3749	6.7208	6.766	6.4513	6.6747
Figure 9	PSNR	–	28.6172	28.7358	28.6396	28.4113	28.5329	28.5645	**28.7356**
	SSIM	–	0.6568	0.6773	0.6715	0.6314	0.6708	0.6585	**0.6841**
	IEP	7.9342	7.4907	7.6095	7.4854	7.6057	7.556	7.5258	*7.4178*
Figure 10	PSNR	–	28.5379	**28.9524**	28.2194	28.3572	28.4469	28.329	28.6159
	SSIM	–	0.4177	0.4322	0.3828	0.3797	**0.45**	0.3762	0.4125
	IEP	7.7544	7.0585	7.0894	6.8253	7.2843	7.0162	6.9766	*6.6345*
Figure 11	PSNR	–	**29.5525**	29.3251	29.2062	29.1356	29.121	29.1267	29.4273
	SSIM	–	**0.5522**	0.5146	0.4872	0.4989	0.5136	0.4754	0.5327
	IEP	7.8756	7.3857	7.3517	*7.1447*	7.4718	7.3656	7.2429	7.3428
Figure 12	PSNR	–	28.9776	29.0562	29.0026	28.8574	29.0278	28.8472	**29.1012**
	SSIM	–	0.492	0.4915	0.4794	0.4826	0.499	0.4408	**0.506**
	IEP	7.3097	6.3323	6.2479	*5.424*	6.4593	6.2646	5.866	6.3496

The information entropy of an image reflects the complexity and uncertainty of information within the image. For similar images, a lower information entropy typically indicates that the information in the image is more concentrated and straightforward. The formula for calculating *IEP* is as follows:

16 $$\begin{aligned} IE=-\sum _{0}^{255} P_i\log _{2}{P_i}, \end{aligned}$$

where $P_i$ represents the probability of gray value *i* appearing in the image. Similar to the use of PSNR and SSIM to measure image quality, when using information entropy to assess the quality of image smoothing, it is important to pay attention not only to the decrease in information entropy, but also to the preservation of the major edges in the original image after smoothing, as the elimination of the edge structure that should have been preserved also results in a decrease in information entropy, and thus the same need to incorporate the visual image as a reference. We separately calculated the information entropy of the images, and the results are summarized in Table 1. The values in italics in the row corresponding to IEP indicate the minimum values in the row. Combining the smoothing results with the data in the table, it can be observed that our method significantly reduces the entropy of the image while effectively preserving the structural edges of the image.

## Conclusion

To conclude, this paper propose an image smoothing method This method combine global gradient sparsity and local relative gradient to obtain more accurate image structure edges. On this basis, this method use the global optimization to smooth the image, avoiding the shortcomings of the local filtering method while retaining the weak edges. We demonstrate the effectiveness of our method for multiple texture styles.

Our method does have some limitations. Compared to some classical methods, our method first decomposes the input image to accurately distinguish between texture and edge. This can achieve a satisfactory smoothing effect, but at the same time, it also increases the algorithm’s running time. The application scenario of our method lies in the pre-processing or post-processing process which does not have strong real-time requirements and has higher requirements for the results.

Our work will continue to expand in two directions. Firstly, we aim to further improve the overall computational speed of our method to accommodate tasks with stricter timing requirements. Secondly, we plan to conduct research on parameter selection to enhance the generality of our method.

## Data Availability

The datasets used and analysed during the current study available from the corresponding author on reasonable request.

## Code availability

The Matlab code of this method available from the corresponding author on reasonable request.
